# Fear but not fright: re-evaluating traumatic experience attenuates anxiety-like behaviors after fear conditioning

**DOI:** 10.3389/fnbeh.2014.00279

**Published:** 2014-08-25

**Authors:** Marco Costanzi, Daniele Saraulli, Sara Cannas, Francesca D’Alessandro, Fulvio Florenzano, Clelia Rossi-Arnaud, Vincenzo Cestari

**Affiliations:** ^1^Cell Biology and Neurobiology Institute (IBCN), CNR/IRCCS Fondazione Santa LuciaRome, Italy; ^2^Department of Human Sciences, Libera Università Maria SS. AssuntaRome, Italy; ^3^Department of Psychology, Sapienza UniversityRome, Italy; ^4^Confocal Microscopy Unit, EBRI-European Brain Research InstituteRome, Italy; ^5^Department of Psychology and “Daniel Bovet” Center, Sapienza UniversityRome, Italy

**Keywords:** contextual fear conditioning, fear sensitization, spontaneous recovery, fear reinstatement, amygdala, lateral orbitofrontal cortex

## Abstract

Fear allows organisms to cope with dangerous situations and remembering these situations has an adaptive role preserving individuals from injury and death. However, recalling traumatic memories can induce re-experiencing the trauma, thus resulting in a maladaptive fear. A failure to properly regulate fear responses has been associated with anxiety disorders, like Posttraumatic Stress Disorder (PTSD). Thus, re-establishing the capability to regulate fear has an important role for its adaptive and clinical relevance. Strategies aimed at erasing fear memories have been proposed, although there are limits about their efficiency in treating anxiety disorders. To re-establish fear regulation, here we propose a new approach, based on the re-evaluation of the aversive value of traumatic experience. Mice were submitted to a contextual-fear-conditioning paradigm in which a neutral context was paired with an intense electric footshock. Three weeks after acquisition, conditioned mice were treated with a less intense footshock (pain threshold). The effectiveness of this procedure in reducing fear expression was assessed in terms of behavioral outcomes related to PTSD (e.g., hyper-reactivity to a neutral tone, anxiety levels in a plus maze task, social avoidance, and learning deficits in a spatial water maze) and of amygdala activity by evaluating c-fos expression. Furthermore, a possible role of lateral orbitofrontal cortex (lOFC) in mediating the behavioral effects induced by the re-evaluation procedure was investigated. We observed that this treatment: (i) significantly mitigates the abnormal behavioral outcomes induced by trauma; (ii) persistently attenuates fear expression without erasing contextual memory; (iii) prevents fear reinstatement; (iv) reduces amygdala activity; and (v) requires an intact lOFC to be effective. These results suggest that an effective strategy to treat pathological anxiety should address cognitive re-evaluation of the traumatic experience mediated by lOFC.

## Introduction

Fear allows organisms to cope with dangerous situations and remembering these situations has an adaptive role preserving individuals from injury and death. However, recalling traumatic memories can induce re-experiencing the trauma, thus resulting in a maladaptive fear. A failure to properly regulate fear responses has been associated with anxiety disorders, like Posttraumatic Stress Disorder (PTSD). Thus, re-establishing the capability to regulate fear is important for its adaptive and clinical relevance (Amstadter, 2008; Hartley and Phelps, 2010; Maren, 2011).

Fear conditioning, in which an initially neutral stimulus (conditioned stimulus, CS e.g., a tone, a light or the conditioning context) is paired with an unconditioned stimulus (US e.g., a shock), is the most widely used experimental paradigm to study emotional associative memories and the neurobiological circuits involved in anxiety disorders (Lissek, 2012). Fear responses (e.g., freezing and increased startle) after a contextual fear conditioning present striking similarities with the symptoms of anxiety; in particular with those aspects of pathological anxiety concerning the feeling of aversive expectation about potential danger (Grillon, 2002).

Extinction, whereby the non-reinforced CS is presented, is the simplest way to regulate fear expression, although its beneficial effect turns out to be temporary and context dependent (Bouton, 2004). Recently, pharmacological and behavioral strategies aimed at erasing fear memory (i.e., facilitating extinction processes) have been proposed (Kindt et al., 2009; Monfils et al., 2009; Schiller et al., 2010). Overall, the rationale of these strategies is to weaken the strength of CS-US association by attenuating the acquired aversion to the CS. However, limits about their efficiency in erasing fear memories and in treating anxiety disorders have been pointed out (Milekic and Alberini, 2002; Quirk and Milad, 2010; Costanzi et al., 2011; Maren, 2011).

Besides the associative CS-US memory, acquisition of conditioned fear is accompanied by a nonassociative sensitization through which an aversive experience leads to a general potentiation of the responsiveness to sensory stimuli that have the capacity to elicit fear, regardless of their context of occurrence (Kamprath and Wotjak, 2004; Siegmund and Wotjak, 2007a; Costanzi et al., 2011). There is evidence that this nonassociative component is dependent on US intensity (Harris, 1943; Kamprath and Wotjak, 2004; Siegmund and Wotjak, 2007a) and largely independent from the associative memory (Gewirtz et al., 1998; Siegmund and Wotjak, 2007b). Moreover, it is causally involved in the onset of abnormal behaviors (e.g., hyperarousal, social withdrawal and learning deficits) characterizing anxiety disorders, such as PTSD (Siegmund and Wotjak, 2007a; Costanzi et al., 2011). As a causative factor in the pathomechanisms of emotional diseases (Shalev et al., 2000; Kamprath and Wotjak, 2004; Siegmund and Wotjak, 2006), fear sensitization emerges as a target of choice for effective behavioral therapeutic interventions.

To re-establish fear regulation, here we propose a new approach based on the hypothesis that the re-evaluation of the aversive value of traumatic experience could reduce fear sensitization and promote the recovery from trauma-induced behavioral abnormalities. To test this hypothesis, we submitted mice to a post-conditioning treatment in which a series of individually determined pain threshold footshocks (PT) was administered in the training context. The choice of PT treatment was based on previous findings showing that: (i) post-conditioning modulation of US representation, by reduced shock intensity, leads to an attenuation of conditioned responses in rats (Rescorla and Heth, 1975) rabbits (Kehoe and White, 2002) and humans (Hosoba et al., 2001); (ii) a PT footshock is able to induce associative fear memory but not sufficient to produce pathological fear sensitization in mice (Siegmund and Wotjak, 2007a; Costanzi et al., 2011).

The effectiveness of PT treatment was investigated in a series of tests (i.e., reaction to a neutral tone, plus maze, social avoidance/approach, spatial water maze) that allow to measure the behavioral abnormalities due to the sensitization process, resembling symptoms in post-traumatic patients (Cohen et al., 2003; Louvart et al., 2005; Kohda et al., 2007; Siegmund and Wotjak, 2007a).

Further experiments were carried out to verify the effectiveness of PT treatment in preventing the return of fear due to spontaneous recovery and fear reinstatement. The activity of amygdala, a brain structure involved in fear expression and anxiety (Beck and Fibiger, 1995; Ehrlich et al., 2009; Knapska and Maren, 2009; Poulos et al., 2009; Ciocchi et al., 2010; Haubensak et al., 2010; Tye et al., 2011; Mahan and Ressler, 2012; Pare and Duvarci, 2012), was also investigated after PT treatment by immunohistochemical analyses of the expression of the early gene *c-fos*.

Finally, the hypothesis that PT treatment reduces fear expression through a re-evaluation process was tested by performing lesions of lateral orbitofrontal cortex (lOFC) before the treatment. Indeed, increasing evidence shows that the lOFC is involved in processing information about the incentive value of unconditioned stimuli and drives behavior in devaluation paradigms, in which conditioned responses after appetitive conditioning are reduced by pairing the appetitive US with a drug-induced illness or by inducing food satiation (Gallagher et al., 1999; Pickens et al., 2003; Delamater, 2007; West et al., 2011, 2013; Wilson et al., 2014).

## Materials and methods

**Subjects:** A total of 216 C57BL/6N male mice (Charles River), aged 8 weeks and weighing 25–30 g were used in these experiments. Upon arrival, mice were housed in groups of four in standard breeding cages (21 × 21 × 12 cm) and kept in a 12-h light/dark cycle (lights were ON from 07:00 to 19:00 h) at a constant temperature of 21°C. Food and water were provided *ad libitum*. All procedures were conducted in accordance to Italian national laws and regulations on the use of animals in research and European guidelines on animal care. Maximum care was taken to minimize the number of animals used and to minimize their suffering.

**Fear Conditioning:** Mice were submitted to a training procedure for contextual fear conditioning (TSE system, Bad Homburg, Germany). In brief, mice were placed in the conditioning chamber A (26 × 22 × 18 cm blue Plexiglas box, 19 lux, cleaned with a 30% v/v of ethanol solution) and after 198 s of acclimation period an electric footshock was delivered (pain threshold, 0.7 mA or 1.5 mA, 2 s) from the metal-grid floor. Animals remained in the shock chamber for another 60 s before they were returned to their home cage.

**Freezing evaluation:** Freezing was defined as the complete absence of voluntary movements, except for respiratory movements. In all experiments, freezing was manually recorded by pressing preset keys (one for the start and one for the end of every freezing phase) on a computer keyboard, using EthoVision software (Noldus Information Technology). Freezing behavior for statistical analysis was quantified by converting the total time freezing into percentage of freezing. All test sessions were video-recorded and the amount of freezing was subsequently scored (offline) by an experimenter blind to behavioral treatments.

**Behavioral Treatments:** Three weeks after conditioning, when remote fear memory was established, animals were treated with either an extinction procedure or a PT exposure. For extinction, mice were placed for 5 days (6 min per day) in the conditioning chamber A without footshock administration. Similarly, mice submitted to the PT treatment were placed in the conditioning chamber A for 5 days (6 min per day), but after the first 3 min a single pain threshold electric footshock was delivered by manually increasing (with steps of 0.1 mA) footshock intensity until the pain threshold was reached, within a maximum period of 2 s. Pain threshold to footshock was defined as the lowest shock intensity at which an animal’s hind foot left the metal floor and individually adjusted on each day of treatment. Two-way ANOVA carried out on footshock intensities recorded in all groups of mice submitted to the PT treatment showed no significant differences (*F*_s_ < 2; *P* > 0.3) in the level of footshock needed to reach the pain threshold.

**Memory test:** Four weeks after training, contextual memory was evaluated by exposing animals to the conditioning chamber A for 3 min.

**Fear sensitization test:** Four weeks after memory test, the reaction to a neutral tone (Tone test) was evaluated by placing mice in a new context B (a cylindrical transparent box of 20 cm in diameter with wood shavings on the floor and lighted with a blue-tensor lamp,18 lux, cleaned with a 1% v/v acetic acid solution) in which a neutral tone (80 dB, 9 kHz) was continuously delivered during the last 3 min of a 6 min period.

**Spontaneous recovery:** Two days after treatments (T1), contextual memory and fear sensitization were evaluated in extinguished and PT-treated mice. Four weeks later (at T2), spontaneous recovery was evaluated by returning animals in the conditioning chamber A for 3 min. The reaction to a neutral tone was again evaluated by placing animals in a different context C (26 × 22 × 18 cm black Plexiglas box with a gray plastic floor lighted with a red-tensor lamp, 57 lux, a lemon essence smell, and cleaned with a 10% v/v ethanol solution) in which a neutral tone (the same used in T1) was continuously delivered during the last 3 min of a 6 min period.

**Fear reinstatement:** Two days after treatment (T1), mice were submitted to the contextual memory test and fear sensitization test (Tone) using the same behavioral procedure presented previously. One week after T1, all mice received an immediate electric footshock (iUS; 0.7 mA, 2 s) in the contextual memory test (CTX) A. The day after the immediate shock (T2), reinstatement was evaluated by placing animals in the conditioning chamber A for 3 min. Fear sensitization was evaluated as in the previous experiment.

**Elevated plus maze test:** Anxiety levels were recorded in an elevated maze consisting of two arms closed by gray walls, 15-cm high, and two open arms.

**Social approach/avoidance behavior test:** This test was carried out in a rectangular box (41 × 70 × 28 cm) divided in three interconnected equal chamber. The test mouse was placed in the central chamber of the apparatus and was allowed to freely explore all three chambers during a 5-min acclimation period (1st session). At the end of the initial 5-min period, the mouse stimulus (a juvenile C57BL/6N male mouse) was placed in one of two cylinders located in the lateral chambers. The time that the test mouse spent in each of the three chambers was again measured over the next 5 min (2nd session). A social avoidance/approach index was calculated as the ratio of the time spent in social and nonsocial chambers according to the formula: *T*_social side_/(*T*_social side_ + *T*_non social side_).

**Morris water maze task:** It was carried out in a circular swimming pool (130 cm of diameter) in which a hidden, 15-cm diameter platform was placed. The training consisted of 18 trials (six trials per day, lasting a maximum of 60 s, with an intertrial interval of 30 min). The start position was changed for each trial with the platform left in the same position. Behavior was evaluated by EthoVision software (Noldus Information Technology).

**Open field test:** Mice were placed in the center of a square (52 × 52 cm) arena and allowed to explore the apparatus for 5 min. Behavior was videotaped by a video camera placed above the apparatus and the time spent near the wall (thigmotaxis), in the center of the apparatus and the distance moved were analyzed by using the Ethovision software (Noldus information Technology).

**Tissue preparation and imaging to asses amygdala *c-fos* activity:** One hour after tests 21 mice were anesthetized with an overdose of chloral hydrate (450 mg/kg, i.p., Fluka) and intracardially perfused with ice-cold phosphate-buffered saline (PBS) followed by 4% paraformaldehyde/PBS (PFA) solution. The brains were removed and stored for 24 h at 4°C in the same fixative. After three washes, brains were immersed in sucrose/PBS 30% solution at 4°C and cryopreserved at −80°C.

Coronal sections (40 μm) from brains embedded in Tissue-Tek OCT (Sakura) were obtained in a cryostat with a temperature of −22°C. Sections (ranging from 1.06 mm to 1.70 mm posterior to bregma) containing the amygdala region were collected and processed for double labeling with cellular markers anti-rabbit c-fos and anti-mouse NeuN (both 1:500; Cell Signaling) using fluorescent methods. Slides were examined with a confocal laser scanning microscope (20× and 10× dry objectives; Leica SP5, Leica Microsystems, Wetzlar, Germany) equipped with three laser lines: violet diode emitting at 405 nm, argon emitting at 488 nm and helium/neon emitting at 543 nm. Confocal acquisition modality was multitrack and all the settings were maintained constant across different cases. For production of figures, a .tiff file was exported, brightness and contrast of images were adjusted and final figures were assembled by using Adobe Photoshop CS5. Amygdala nuclei (lateral, basal, centrolateral, centromedial) were identified using a mouse brain atlas and by assessing nuclei boundaries through NeuN staining. Only c-fos positive cells, which were also NeuN positive, were counted bilaterally according to a Region Of Interest (ROI) procedure. Four sections for mouse (160 μm apart), covering the whole rostro-caudal extent of the amygdala, were analyzed to count cells expressing the indicated markers. Only cells that expressed colocalization were analyzed. Cells were considered colocalized when they expressed both red (NeuN+) and Green (c-fos+) fluorescence. The total estimated number of cells within each region expressing c-fos/NeuN colocalization was obtained by dividing the number of c-fos/NeuN positive cells for the entire area of interests expressed in mm^2^. Measurements of positive cells and areas were obtained by computer-assisted analysis using the ImageJ software.

**Orbitofrontal cortex lesions:** Two weeks after training mice were anesthetized with Chloral Hydrate (400 mg/Kg, i.p., Fluka) and placed in a stereotaxic apparatus (David Kopf Instrument with mouse adaptor). Ibotenic acid (1 mg in 200 μl of PBS; Sigma-Aldrich) was bilaterally infused into the lateral and ventral orbital cortices (LO and VO respectively) in a volume of 0.25 μL with a rate of 0.1 μL/min at the following stereotaxic coordinates: AP = +2.5; ML = ±1.2; DV = −2.8 (Franklin and Paxinos, 1997). After surgery, mice were allowed 1 week to recover before behavioral experiments. At the end of behavioral experiments the localization and extension of damaged tissue were analyzed through cresyl violet staining.

**Statistical Analyses:** The statistics were based on one-, two- or three-way ANOVA and between-group comparisons using Duncan’s test. *P* < 0.05 was considered significant.

## Results

### Effects of PT treatment on fear expression and anxiety-like symptoms after fear conditioning

**Figure 1:**
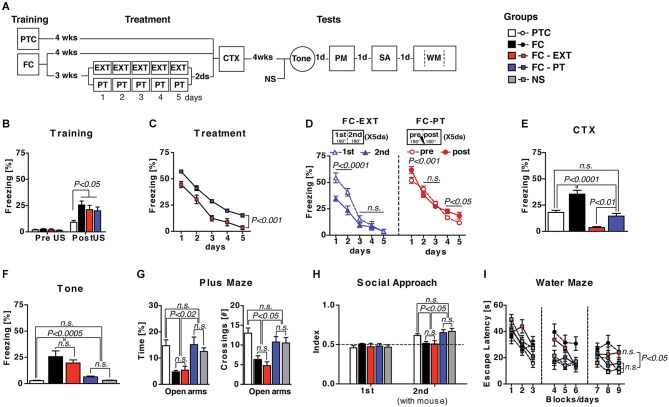
**PT treatment restores normal fear regulation**. (**A**) Experimental schedule and groups. FC, fear conditioned mice (*n* = 9); PTC, painthreshold footshock conditioned mice (*n* = 9); FC-EXT, fear conditioned mice submitted to the extinction treatment (*n* = 8); FC-PT, FC mice submitted to painthreshold footshock treatment (*n* = 8); NS, no shocked mice (*n* = 8). (**B**) Freezing (%) during the training before footshock administration. (**C**) Five days of treatment reduced freezing (%) in both FC-PT and FC-EXT, although the latter group froze significantly less than the former. (**D**) Freezing (%) during each daily trial (from day 1 to day 5) in FC-EXT (*left*) and FC-PT (*right*) mice, separately analyzed considering the first 180” (1st) and the second 180” (2nd) in extinguished mice and the 180” before (pre) and after (post) PT administration in PT-treated mice. The different patterns of freezing reduction induced by the two treatments suggest that freezing decrease induced by PT treatment is not due to extinction. (**E)** Freezing (%) in the contextual memory test (CTX). PT treatment dampened contextual fear without erasing memory. (**F**) Freezing (%) in response to a neutral tone (Tone) in a new context. Fear sensitization decreased in FC-PT, but not in FC-EXT. (**G**) Time spent in (*left*) and the number of crossing to (*right*) open arms in an elevated plus maze task. (**H**) Social interaction [index: *T*_social side_/(*T*_social side_ + *T*_non social side_)] in a social avoidance/approach task. (**I**) Escape latencies (s) in the spatial water maze. PT treatment, but not extinction, improved anxiety behaviors, social withdrawal and spatial learning deficits induced by fear conditioning. Data are expressed as mean + SEM. Results of individual *post hoc* comparisons (Duncan’s and Fisher’s tests) are depicted in the figure [n.s., not significant = *P* > 0.05].

Fear conditioned (FC) mice were treated with a standard extinction procedure (FC-EXT) or with a pain threshold footshock (FC-PT), in which the shock intensity was individually determined on each day during the actual treatment and never exceeded the intensity of 0.4 mA. After treatments, mice were submitted to a series of tests in order to evaluate memory retention and abnormal behavior induced by a traumatic experience. Three additional groups of FC, pain threshold footshock conditioned (PTC) or non-shocked (NS) mice were tested as additional controls (Figure 1A). Three weeks after training, when a remote fear memory is established, mice conditioned with the high footshock (0.7 mA) were exposed to standard extinction (EXT), PT-treatment (PT) or left undisturbed (FC). The analysis of freezing recorded after footshock administration during the training (Figure 1B) revealed a significant effect of shock intensity (one-way ANOVA, *F*_(3,30)_ = 4.45; *P* < 0.02). During treatment (Figure 1C), FC-PT and FC-EXT mice exhibited a reduction in freezing (*F*_(4,56)_ = 292.96; *P* < 0.0001), although FC-EXT mice froze significantly less than FC-PT mice (*F*_(1,14)_ = 30.19; *P* < 0.0002). A detailed analysis of the freezing levels in FC-EXT and FC-PT mice revealed differences in the pattern of freezing reduction induced by the two treatments, suggesting that freezing decrease induced by PT treatment is not due to extinction (Figure 1D). To this aim freezing levels in both FC-EXT and FC-PT were separately analyzed (two-way ANOVAs) considering the first vs. the second half of each daily trial, that in the case of FC-PT refers to the periods preceding and following PT administration, respectively. The results obtained in extinguished mice (Figure 1D, left) showed significant effects of *period* (*F*_(1,14)_ = 6.15; *P* < 0.05), *days* (*F*_(4,56)_ = 115.77; *P* < 0.0001) and *period* × *days* interaction (*F*_(4,56)_ = 8.12; *P* < 0.00001). A within-day analysis (Duncan’s test) between freezing levels in 1st and 2nd revealed significant differences on the first (*P* < 0.001) and second day (*P* < 0.001), but not on the third (*P* = 0.07), fourth (*P* = 0.67) and fifth day (*P* = 0.86). A between-day analysis revealed a significant freezing decrease from day 1 to day 5 (*P* < 0.00003). These data indicated that freezing reduction in FC-EXT mice was due to a within- and a between-trial extinction. Conversely, the results obtained in PT-treated mice (Figure 1D, right) showed no significant effect of *period* (*F*_(1,14)_ = 1.47; *P* = 0.2), but significant effects of both *days* (*F*_(4,56)_ = 163.48; *P* < 0.0001) and *period* × *days* interaction (*F*_(4,56)_ = 6.17; *P* < 0.001). A within-day analysis (Duncan’s test) revealed a significant increase in freezing levels after PT administration (pre vs. post) on the first (*P* < 0.01), fourth (*P* < 0.0001) and fifth (*P* < 0.001) day of treatment. No significant differences were observed on the second (*P* = 0.06) and third day (*P* < 0.41). Instead, a between-day analysis revealed a significant freezing decrease from day 1 to day 5 (*P* < 0.0001). These results indicated that freezing reduction induced by PT treatment was only due to a between-trial effect.

Contextual memory test (CTX; Figure 1E) revealed significant differences among groups (*F*_(3,30)_ = 26.59; *P* < 0.0001) with FC-EXT mice showing the lowest level of freezing in comparison with all other groups (*P* < 0.001), while FC-PT mice were comparable to PTC mice (*P* > 0.05) but froze significantly less than FC mice (*P* < 0.001). In the Tone test (Figure 1F), in which the reaction to a neutral tone in a new context is considered as a measure of fear sensitization (Costanzi et al., 2011), a significant difference among groups (*F*_(4,37)_ = 13.18; *P* < 0.0001) was observed, with FC-PT, PTC and NS mice showing similar lower levels of freezing when compared to FC-EXT and FC mice. Analogous results (*F*s > 4; *P* < 0.005) emerged when anxiety levels, social interaction and spatial learning were analyzed (Figures 1G–I). Overall, the performance of FC-PT mice was comparable to that of PTC and NS mice, while FC-EXT and FC mice showed the same behavioral deficits. Taken together, these results indicate that PT treatment reduces contextual fear memory without erasing it and prevents behavioral abnormalities related to the traumatic memory.

### Freezing reduction induced by PT treatment after conditioning is not due to an extinction-like process

To better ascertain whether freezing reduction induced by PT treatment in fear-conditioned mice was not due to an extinction-like process we performed two further experiments.

**Figure 2:**
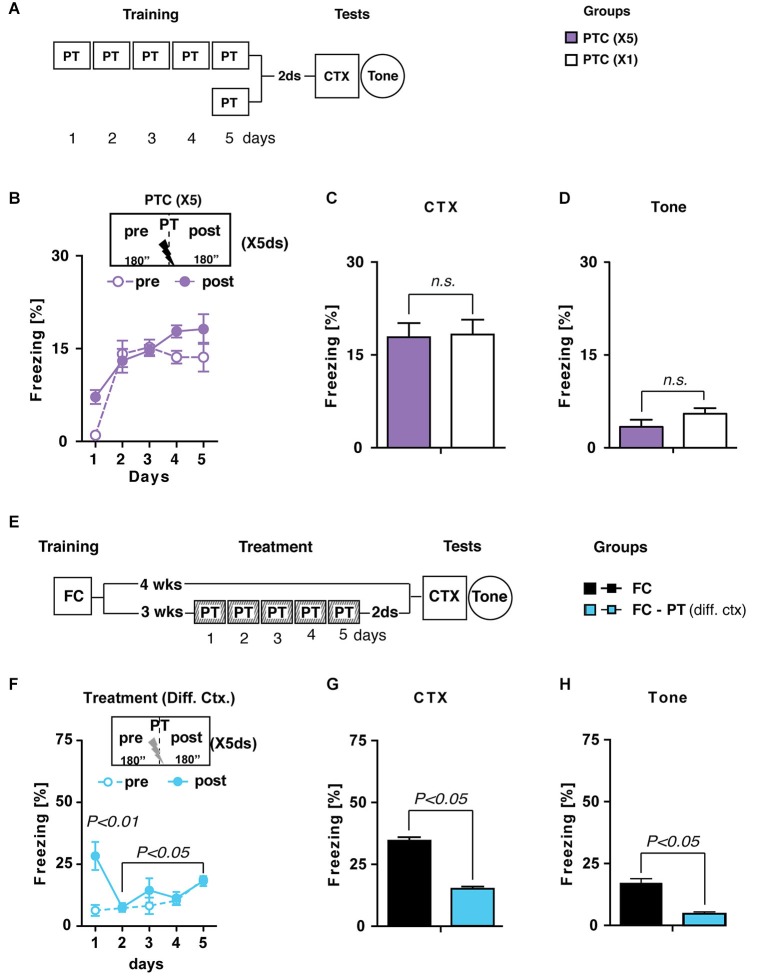
**PT treatment is different from extinction**. (**A**) Experimental schedule and groups. PTC (X5), mice trained for five consecutive days (6 min/day) with a painthreshold footshock (PT) administered when the first 180” were elapsed (*n* = 8); PTC (X1), mice trained with a PT in a standard training protocol (*n* = 6). (**B**) Freezing (%) during PTC (X5) training was plotted as a mean of the 180” before (pre) and after (post) PT administration for each daily trial. (**C**) Freezing (%) recorded during CTX carried out 2 days after PTC (X1) and PTC (X5). (**D**) Freezing (%) recorded in reaction to a neutral tone (Tone) administered in a new context. Together, these results indicated that PTC (X5) was able to increase freezing at levels comparable with those observed in PTC (X1) mice trained with the standard protocol and it did not induce fear sensitization. (**E**) Experimental schedule and groups. FC, fear-conditioned mice (*n* = 8) submitted to contextual memory test (CTX) and fear sensitization (Tone) 4 weeks after training. FC-PT_(diff. ctx)_, fear-conditioned mice (*n* = 8) treated with the PT administration in a different context 3 weeks after training and then submitted to CTX and Tone. (**F**) Freezing (%) during PT treatment in a different context (FC-PT_(diff. ctx)_) was plotted as a mean of the 180” before (pre) and after (post) the PT administration for each daily trial. (**G–H**) Freezing (%) in the (**G**) CTX and (**H**) in response to a neutral tone (Tone) recorded in FC and FC-PT_(diff. ctx)_. The results indicate that PT treatment carried out in a different context was able to reduce both contextual fear memory and fear sensitization. Data are expressed as mean + SEM. Results of individual *post hoc* comparisons (Duncan’s and Fisher’s tests) are depicted in the figure [n.s., not significant = *P* > 0.05].

In the first experiment (Figure 2A), mice were trained for five consecutive days (6 min/day) with a pain threshold footshock (PTC_(X5)_) and tested for contextual memory retention, as well as for fear sensitization, 2 days later. A second group of mice submitted to a standard one-trial training with a pain threshold footshock (PTC_(X1)_) was considered as a control group. We hypothesized that if PT treatment is an extinction-like process then mice submitted to this procedure, without any previous conditioning, should display a freezing decrease during the treatment. Statistical analysis (two-way ANOVA; Figure 2B) performed considering *period* (pre and post PT administration) as between factor and *days* (1–5) as within factor showed no significant effect of *period* (*F*_(1,14)_ = 2.9; *P* > 0.05) but significant effects of both *days* (*F*_(4,56)_ = 30.16; *P* < 0.0001) and *period* × *days* interaction (*F*_(4,56)_ = 3.22; *P* < 0.05). Individual *post hoc* comparisons (Duncan’s test) revealed that freezing significantly increased after the first day of training (*P* < 0.005) remaining stable during the following 3 days. Contextual memory retention and fear sensitization (Figures 2C,D) of PT(_X5_) mice were undistinguishable from those of PT(_X1_) mice (one-way ANOVAs: *F*_s_ < 2; *P* > 0.05), indicating that PT treatment *per se* induces a non-traumatic fear memory.

In the second experiment (Figure 2E) two groups of FC mice were submitted to either a PT treatment in a new context (FC-PT_(diff. ctx)_) 3 weeks after conditioning or left undisturbed until contextual memory and fear sensitization tests were carried out 4 weeks after training. Since extinction is a context-dependent process (Bouton, 2004), we expected that if PT treatment reduces fear expression through an extinction-like process then it should be effective only when administered in the same context in which traumatic experience occurred.

Statistical analysis (two-way ANOVA) carried out on freezing levels recorded during PT treatment in a different context revealed a significant effect of the treatment (*F*_(4,56)_ = 6.46; *P* < 0.0005) with mice showing a gradual increase of freezing during the 5 days. No significant differences were observed between the amount of freezing recorded before and after PT administration (*F*_(1,14)_ = 4.48; *P* > 0.05). Notably, the negligible amount of freezing on day 1 before PT administration indicated that generalization did not occur (Figure 2F).

During contextual memory (Figure 2G) and fear sensitization (Figure 2H) tests FC-PT_(diff. ctx)_ mice froze significantly less (one-way ANOVAs: *F* > 1; *P* < 0.05) than FC mice, indicating that PT treatment carried out in a novel context, different from that used for the original conditioning, is still able to attenuate fear expression due to a traumatic experience.

Together these results strongly support the above reported findings (see Figure 1D) about the difference between extinction and PT treatment, ruling out the possibility that the effects of PT treatment after fear conditioning were due to an extinction-like process.

### PT treatment prevents spontaneous recovery and reduces fear reinstatement

**Figure 3:**
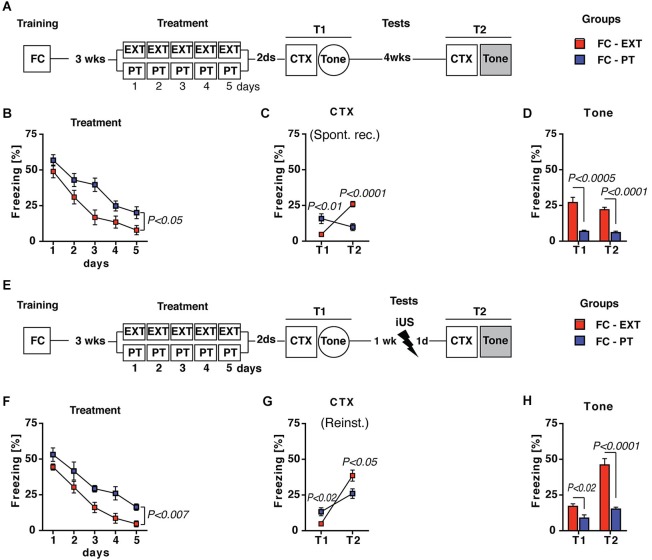
**PT treatment prevents spontaneous recovery and reinstatement**. (**A**) Experimental schedule for spontaneous recovery evaluation. FC-EXT (*n* = 8) and FC-PT (*n* = 8) were submitted to contextual memory (CTX) and fear sensitization tests 2 days after treatment (T1). Spontaneous recovery was evaluated 4 weeks after T1 by exposing FC-EXT and FC-PT mice to the CTX (T2). Fear sensitization was again evaluated exposing animals to a new context with tone presentation (Tone, T2). (**B**) Five days of treatment reduced freezing (%) in both groups, although FC-EXT showed the lower levels of freezing (**C**). Freezing (%) in CTX carried out 2 days after treatment (T1) and 4 weeks later (T2). The results indicate that spontaneous recovery was prevented in FC-PT mice. (**D**) Freezing (%) in response to a neutral tone (Tone) in a new context at both T1 and T2. Prevention of fear sensitization was persistent in FC-PT mice. (**E**) Experimental schedule for reinstatement evaluation after an immediate footshock (iUS = 0.7 mA × 2 s) administered 1 week after treatment. FC-EXT (*n* = 8) and FC-PT (*n* = 8) were submitted to contextual memory (CTX) and fear sensitization (Tone) tests before (T1) and after (T2) iUS administration. (**F**) Five days of treatment reduced freezing (%) in both FC-EXT and FC-PT mice, with FC-EXT showing the lower levels of freezing. (**G–H**) Freezing (%) in (**G**) CTX and in (**H**) response to a neutral tone (Tone) recorded 2 days after treatment (T1) and the day after (T2) iUS administration. PT treatment attenuate reinstatement and fear sensitization after iUS. Data are expressed as mean + SEM. Results of individual *post hoc* comparisons (Duncan’s and Fisher’s tests) are depicted in the figure [n.s., not significant = *P* > 0.05].

Two additional assays were carried out in order to evaluate the effectiveness of PT treatment in preventing the return of fear in spontaneous recovery and reinstatement. Both appear after extinction, the former due to the mere passage of time, the latter to an unsignaled US exposure (Bouton, 2004). Spontaneous recovery was assessed in FC-PT and FC-EXT mice submitted to CTX 4 weeks (T2) after treatment (Figure 3A). Statistical analysis (two-way ANOVA) revealed that 5 days of treatment (Figure 3B) reduced (*F*_(4,56)_ = 154.85; *P* < 0.0001) freezing in both FC-EXT and FC-PT, although FC-EXT showed the lower levels of freezing (*F*_(1,14)_ = 5.47; *P* < 0.05).

In the CTX carried out 2 days (T1) and 4 weeks (T2) after treatments statistical analysis (two-way ANOVA) revealed significant differences (*F*s > 5; *P* < 0.05) between FC-EXT and FC-PT mice. In particular, FC-EXT mice showed lower levels of freezing than FC-PT mice in the contextual memory test carried out 2 days after treatment (Figure 3C, T1, *P* < 0.01), indicating an effective consolidation of extinction. However, spontaneous recovery was prevented in FC-PT, but not in FC-EXT mice when contextual memory test was repeated 4 weeks later (Figure 3C, T2, *P* < 0.0001). Fear sensitization (Tone; Figure 3D) development, tested both 2 (T1) and 30 (T2) days after treatment, was also prevented in FC-PT mice (one-way ANOVAs, *F*s > 25; *P* < 0.001). For the reinstatement experiment (Figure 3E), conditioned mice were treated with extinction (FC-EXT) or with PT procedure (FC-PT) and exposed to an immediate unsignaled footshock (iUS) 1 week after T1 tests. Fear reinstatement was evaluated the next day in both CTX and Tone tests (T2). Also in this case the statistical analysis (two-way ANOVA) revealed that the 5 days of treatment (Figure 3F) reduced (*F*_(4,56)_ = 51.89; *P* < 0.0001) freezing in both FC-EXT and FC-PT, with FC-EXT showing the lower levels of freezing (*F*_(1,14)_ = 10.87; *P* < 0.01). Results obtained in CTX (Figure 3G) and Tone (Figure 3H) tests (two- and one-way ANOVAs, *F*_s_ > 12; *P* < 0.005 and *F*_s_ > 8; *P* < 0.05, respectively) showed higher levels of freezing after the reinstatement procedure in FC-EXT compared to FC-PT (*P* < 0.0001). Together, these results indicate that PT treatment is able to prevent spontaneous recovery and to attenuate reinstatement.

### PT treatment is able to reduce fear expression and anxiety-like symptoms also after a FC training in which animals experience higher footshock

**Figure 4:**
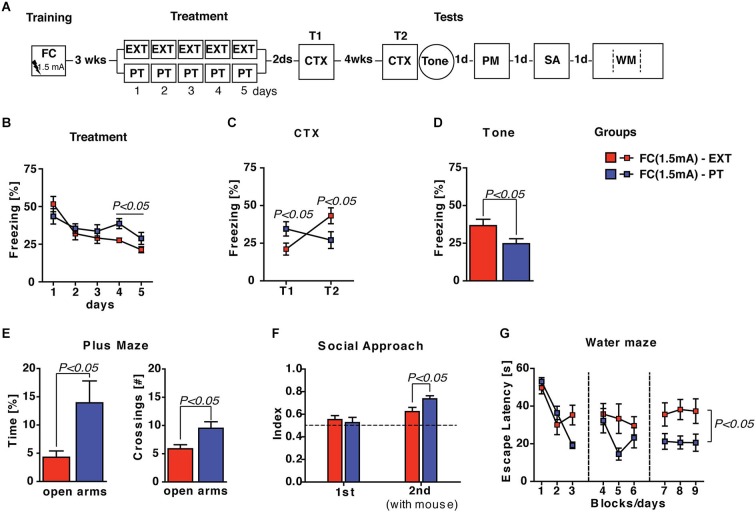
**PT treatment is able to restore emotional regulation also after a FC training with a higher footshock (1.5 mA) administration**. (**A**) Experimental schedule and groups. FC(1.5 mA)-EXT, mice (*n* = 8) underwent 5 days of extinction 3 weeks after fear conditioning employing a higher intensity (1.5 mA) footshock administration; FC(1.5 mA)-PT, mice (*n* = 8) underwent to 5 days of PT treatment 3 weeks after fear conditioning employing a higher intensity (1.5 mA) footshock administration. (**B**) Freezing (%) recorded during the 5 days of treatment with either extinction protocol or PT administration. Freezing decreased in both FC(1.5 mA)-EXT and FC(1.5 mA)-PT mice, although the former froze less than the latter during the last 2 days of treatment. (**C**) Freezing (%) recorded in the contextual memory tests (CTX) carried out 2 days (T1) and 4 weeks (T2) after treatment. Freezing increase in FC(1.5 mA)-EXT mice but not in FC(1.5 mA)-PT mice, indicating that fear spontaneously recovered after extinction but not after PT treatment. (**D**) Freezing (%) in response to a neutral tone (Tone) in a new context. PT treatment was able to reduce fear sensitization after conditioning. (**E**) Time spent in (*left*) and the number of crossing to (*right*) open arms in an elevated plus maze task. Anxiety behaviors were ameliorated after PT treatment. (**F**) Social interaction [index: *T*_social side_/(*T*_social side_ + *T*_non social side_)] in a social avoidance/approach task. FC(1.5 mA)-PT mice spent more time in the social compartment of the apparatus where a mice stimulus was placed in the second session (2nd) of the test, indicating that PT treatment improved the social withdrawal induced by fear conditioning. (**G**) Escape latencies (s) in the spatial water maze. Results indicated that both groups of mice were able to acquire the platform position during the learning, although FC(1.5 mA)-PT mice showed the best performance. Data are expressed as mean + SEM. Results of individual *post hoc* comparisons (Duncan’s and Fisher’s tests) are depicted in the figure [n.s., not significant = *P* > 0.05].

To investigate if the effectiveness of PT treatment in reducing pathological fear is independent from trauma intensity, we decided to increase the footshock intensity (US = 1.5 mA) during the training carried out 3 weeks before extinction and PT treatment. Spontaneous recovery, fear sensitization, anxiety levels, social avoidance and spatial learning were investigated after treatments (Figure 4A). Statistical analysis (two-way ANOVA) of freezing recorded during the 5 days of treatment with either extinction protocol or PT administration revealed significant effects of the *days* factor (*F*_(4,56)_ = 16.98;* P* < 0.0001) and *group* × *days* interaction (*F*_(4,56)_ = 3.29;* P* < 0.005) but no significant effect of the *group* factor (*F*_(1,14)_ = 0.86;* P* > 0.05). Individual *post hoc* comparisons (Duncan’s test) revealed that freezing significantly (*P* < 0.0001) decreased in both FC(1.5 mA)-EXT and FC(1.5 mA)-PT mice, although the former froze significantly (*P* < 0.05) less than the latter during the last 2 days of treatment (Figure 4B).

Contextual memory tests (CTX) carried out 2 days (T1) and 4 weeks (T2) after treatments revealed: (i) an effective consolidation of extinction in FC(1.5 mA)-EXT mice at T1 and; (ii) a spontaneous recovery of fear in FC(1.5 mA)-EXT mice, but not in FC(1.5 mA)-PT mice, at T2 (Figure 4C). Statistical analysis (two-way ANOVA) showed a no significant effect of *group* factor (*F*_(1,14)_ = 0.04;* P* > 0.05) but significant effects of both *time* (*F*_(1,14)_ = 17.83;* P* < 0.001) and *group* × *time* interaction (*F*_(1,14)_ = 71.98;* P* < 0.0001). Individual *post hoc* comparisons (Duncan’s test) revealed a significant freezing increase in FC(1.5 mA)-EXT mice (T1 vs. T2; *P* < 0.0001) and a significant freezing decrease in FC(1.5 mA)-PT mice (T1 vs. T2; *P* < 0.0001), as well as significant between-group differences at both T1 (*P* < 0.05*)* and T2 (*P* < 0.05).

As concerns fear sensitization evaluated as reaction to a neutral tone (Tone) in a new context (Figure 4D), significant differences (one-way ANOVA, *F*_(1,14)_ = 4.94;* P* < 0.05) between FC(1.5 mA)-EXT and FC(1.5 mA)-PT mice were observed, indicating that PT treatment was able to reduce fear sensitization after conditioning carried out with a higher footshock. Similarly, significant differences between the two groups (one- and two-way ANOVAs, *F*s > 5; *P* < 0.05) were observed in plus maze, social avoidance/approach and water maze tasks (Figures 4E–G), indicating that PT treatment after a traumatic experience exerts beneficial effects on anxiety, social avoidance and spatial learning deficits. Altogether, these results showed that PT treatment was still able to prevent spontaneous recovery, to reduce fear sensitization and to ameliorate behavioral abnormalities in mice submitted to a more intense traumatic experience.

### PT treatment after conditioning reduces retrieval-induced expression of c-fos in amygdala

**Figure 5:**
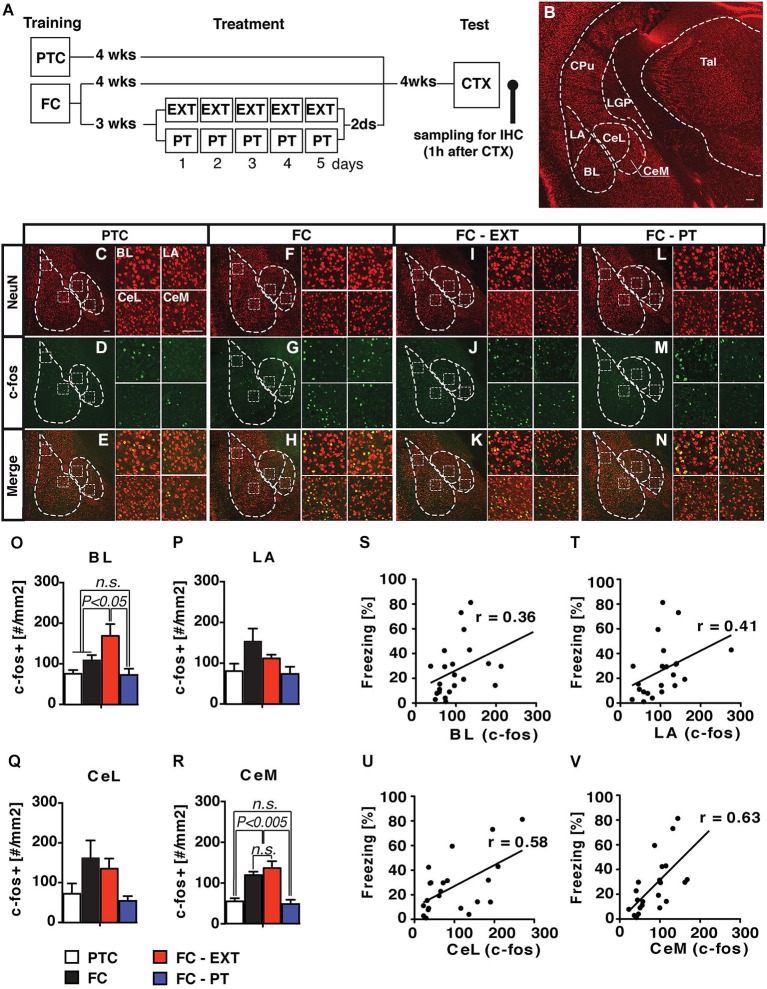
**PT treatment downregulates the activity of amygdala**. (**A**) Experimental schedule and groups. (**B**) Coronal section of the mouse brain showing the anatomical boundaries of the basolateral (BLA) and central (CEA) amygdala. LA/BL, lateral/basal subdivision of BLA; CeL/CeM, lateral/medial subdivisions of CEA. (**C–N**) Confocal microscopy of a double immunofluorescence for NeuN (red) and c-fos (green) in amygdala nuclei of PTC (**C–E**), FC (**F–H**), FC-EXT (**I–K**) and FC-PT (**L–N**) mice submitted to the CTX 4 weeks after treatment. BL, LA, CeL and CeM high magnification insets box. Scale bars = 100 μm. (**O–R**) Analyses of c-fos labelled neurons (#/mm^2^) for each experimental group (PTC, *n* = 7; FC, *n* = 5; FC-EXT, *n* = 4; FC-PT, *n* = 5). (**S–V**) Regression analysis between freezing during CTX and the number of c-fos labeled neurons for each amygdala subdivision. Vertical bars represent SEM. Results of individual *post hoc* comparisons (Duncan’s and Fisher’s tests) are depicted in the figure [n.s., not significant = *P* > 0.05].

We assessed the possible relationship between the behavioral effects of PT treatment and changes in the activity of the amygdala, whose role in fear expression, extinction and the development of pathological anxiety is widely recognized (Tye et al., 2011; Mahan and Ressler, 2012; Pare and Duvarci, 2012). Although amygdala is a complex structure composed by 13 sub-nuclei (Pitkänen et al., 1997), four sub-nuclei (lateral, LA; basal, BL; centrolateral, CeL and centromedial, CeM) are mainly involved in fear expression. In particular, BL and LA are involved in the storage of remote contextual fear memory and extinction (Ehrlich et al., 2009; Poulos et al., 2009), while the CeL and CeM play a major role in regulating the expression of fear responses (Ciocchi et al., 2010; Haubensak et al., 2010). Interestingly, in the latter nuclei, fear memory recall and renewal after extinction seem to be related to an increased number of neurons labeled with c-fos, a marker of neuronal activity (Beck and Fibiger, 1995; Knapska and Maren, 2009). Thus, we investigated amygdala activity by assessing the induction of the early gene *c-fos* in FC-PT or FC-EXT mice exposed to CTX test 4 weeks after treatment, when fear spontaneously recovers in extinguished mice. Two additional groups of control mice (FC and PTC) were exposed to CTX 8 weeks after training (Figure 5A). Amygdala nuclei boundaries were identified through the staining of neuronal marker NeuN (Figure 5B). Only c-fos positive cells (c-fos^+^), which were also NeuN positive, were counted bilaterally according to a ROI procedure (Figures 5C–N). The number of c-fos^+^ neurons (Figures 5O–R) was significantly different among groups in CeM (*F*_(3,17)_ = 17.85; *P* < 0.001) and BL (*F*_(3,17)_ = 7.15; *P* < 0.05), while in CeL and LA there was a trend toward a significant effect (*F*_(3,17)_ = 2.93; *P* = 0.06 and *F*_(3,17)_ = 2.89; *P* = 0.06, respectively). In particular, FC-EXT and FC mice displayed a higher number of c-fos^+^ neurons compared to FC-PT (*P* < 0.002) and PTC (*P* < 0.005) in CeM. A similar pattern of differences was observed also in CeL and LA. In BL, FC-EXT mice exhibited the higher number of c-fos^+^ neurons compared to all other groups (*P* < 0.05). Regression analyses (Figures 5S–V) showed higher correlations between freezing levels and the activities of CeM (*r* = 0.63; *P* < 0.005) and CeL (*r* = 0.58; *P* < 0.01) compared to those between freezing and the activities of BL (*r* = 0.36, *P* > 0.05) and LA (*r* = 0.41; *P* > 0.05). These results indicate that fear reduction and spontaneous recovery prevention induced by PT treatment correlate with the downregulation of neuronal activity into the central amygdala.

### Lateral orbitofrontal cortex (lOFC) mediates the effect of PT treatment

**Figure 6:**
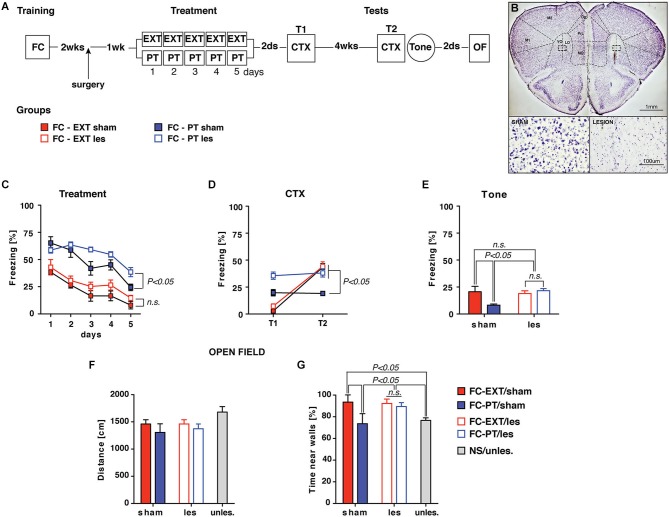
**lOFC lesion blocks the effect exerted by PT treatment on re-establishing fear regulation**. (**A**) Experimental schedule and groups. Two weeks after conditioning mice underwent to a surgical procedure (arrow) in which lesion of lateral orbitofrontal cortex (lOFC, coordinates from bregma: AP = +2.5; ML = ±1.2; DV = −2.8) was performed by ibotenic acid (1.25 μg) administration. One-week later sham and lesion mice were assigned to either EXT or PT treatment, thus four experimental groups were formed: FC-EXT/sham (*n* = 5), FC-EXT/les (*n* = 10), FC-PT/sham (*n* = 6), FC-PT/les (*n* = 9). (**B**) Representative images of Nissl-stained sections of sham (top, left) and lOFC lesion (top, right). Inset boxes represent the area for high magnification of healthy (bottom, left) and damaged (bottom, right) tissue. Note the neuronal loss in the damaged tissue compared to the healthy one. VO: ventral orbital; LO: lateral orbital. (**C**) Five days of treatment reduced freezing (%) in all groups of mice, although FC-PT/les was less sensitive to the effect induced by PT treatment. (**D**) Freezing (%) in contextual memory test (CTX) carried out 2 days (T1) and 4 weeks (T2) after treatment. lOFC lesions block the spontaneous recovery prevention exerted by the PT treatment. (**E**) Freezing (%) in response to a neutral tone (Tone) in a new context. lOFC lesions block the reduction of fear induced by the PT treatment. (**F**) Distance (cm) travelled by animals in an open field. No significant differences among groups were observed, indicating that lOFC lesions did not affect locomotor activity. (**G**) Time (%) spent by animals near walls in the open field. FC-PT mice with lesions of lOFC spent more time near walls when compared with FC-PT/sham and NS/unles. mice, indicating that lOFC lesions reverted the beneficial effect of PT treatment on the reduction of an anxiety-like behavior. Data are expressed as mean + SEM. Results of individual *post hoc* comparisons (Duncan’s tests and Fisher’s tests) are depicted in the figure [n.s., not significant = *P* > 0.05].

This experiment was designed to investigate the mechanism through which PT treatment restored fear regulation, hypothesizing that the efficiency of PT treatment in reducing fear expression could be due to a re-evaluation of the traumatic experience based on US aversiveness devaluation. The devaluation process is well known to reduce the conditioned response after an appetitive conditioning through the activity of the lOFC (Gallagher et al., 1999; Pickens et al., 2003, 2005; West et al., 2011, 2013; Wilson et al., 2014). To this aim FC mice were submitted to lOFC lesion 1 week before PT treatment and extinction (Figures 6A–B). Lesions of lOFC antagonized the effects exerted by PT treatment on freezing reduction recorded during the treatment (three-way ANOVA, *F*s > 4; *P* < 0.05 followed by Duncan’s test for the appropriate *post hoc* analysis), but had no effects on extinction (Figure 6C). Two days (T1) and 4 weeks (T2) after treatment spontaneous recovery was assessed by CTX; fear sensitization (Tone) was assessed after CTX test at T2. The analyses of freezing recorded during both CTX (Figure 6D; three-way ANOVA, *F*s > 6; *P* < 0.05 followed by Duncan’s test for the appropriate *post hoc* analysis) and Tone tests (Figure 6E; two-way ANOVA, *F*s > 4; *P* < 0.05 followed by Duncan’s test for the appropriate *post hoc* analysis), revealed that lOFC lesion completely reverted the beneficial effects exerted by PT treatment on spontaneous recovery prevention and fear sensitization reduction.

To investigate possible non-specific effects of lOFC lesions on locomotor activity all mice were submitted to an open field task (Figure 6F). A further group of naïve mice (NS/unles) was considered as control. No significant differences (one-way ANOVA, *F*_(4,35)_ = 1.59; *P* > 0.05) among groups were observed in the distance travelled in the open field. However, significant differences (one-way ANOVA, *F*_(4,35)_ = 3.52; *P* < 0.05) among groups were observed in respect to the time spent by animals near walls (Figure 6G); such a measure is considered as an index of anxiety (Prut and Belzung, 2003). *Post hoc* analysis (Fisher’s Test) revealed that FC-PT/sham and NS/unles mice spent significantly (*P* < 0.05) less time near walls in comparisons with FC-PT/les and FC-EXT (both sham and lesioned), confirming that the beneficial effect exerted by PT treatment on anxiety reduction was reverted by lOFC lesion.

These results indicate that PT treatment induces a re-evaluation of the US aversiveness, modifying the internal representation of the US. This interpretation is in agreement with previous results in human participants showing that a US deflation procedure is able to decrease the US aversiveness (Hosoba et al., 2001).

### The effects of PT treatment on fear expression are not due to the US habituation and/or expectancy

**Figure 7:**
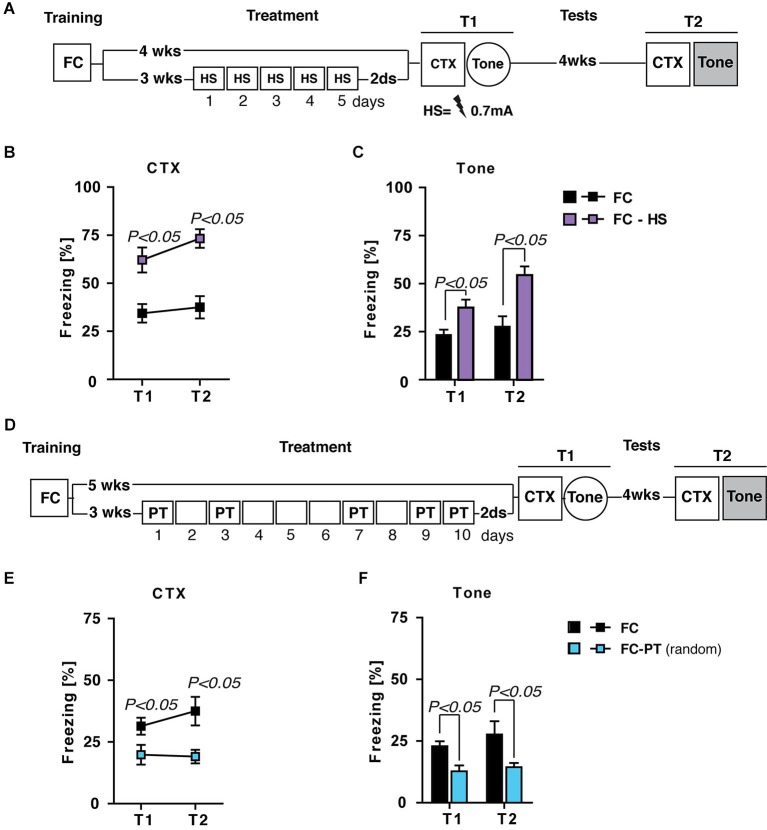
**The effects of PT treatment on fear expression are due neither to US habituation nor US expectancy**. (**A**) Experimental schedule and groups. FC, fear-conditioned mice (*n* = 8) submitted to contextual memory test (CTX) and fear sensitization (Tone) 4 (T1) and 8 (T2) weeks after training. FC-HS, fear-conditioned mice (*n* = 8) treated with a high footshock (0.7 mA; HS) 3 weeks after training and then submitted to CTX and Tone at T1 and T2. (**B**) Freezing (%) in the CTX recorded at T1 and 4 weeks later at T2. Results indicated that HS treatment increased contextual fear memory. (**C**) Freezing (%) in response to a neutral tone (Tone) recorded at T1 and T2. Results indicated that HS treatment increased fear sensitization. (**D**) Experimental schedule and groups. FC, fear-conditioned mice (*n* = 8) submitted to CTX and fear sensitization (Tone) 5 (T1) and 9 (T2) weeks after training. FC-PT_(random)_, fear-conditioned mice (*n* = 7) exposed to PT treatment (10 days) with a random PT administration (on day 1,3,7,9,10) 3 weeks after training and then submitted to CTX and Tone at T1 and T2. (**E**) Freezing (%) in the CTX recorded at T1 and 4 weeks later at T2. The results indicated that PT treatment with a random footshock administration decreased fear in the contextual memory test. (**F**) Freezing (%) in response to a neutral tone (Tone) recorded at T1 and T2. Results indicated that PT treatment with a random footshock administration was able to persistently reduce fear sensitization. Data are expressed as mean + SEM. Results of individual *post hoc* comparisons (Duncan’s tests and Fisher’s tests) are depicted in the figure [n.s., not significant = *P* > 0.05].

Although lOFC lesion results support the idea that the beneficial effects of PT treatment are mediated by the re-evaluation of US aversiveness, alternative explanations have to be taken into account. The first one regards the possibility that repeated exposure to the US during PT treatment could induce a footshock habituation. If so, we expected that the effect of treatment should be independent from US intensity. In order to evaluate this possibility, FC mice were submitted to a treatment in which footshock was daily administered at a prefixed intensity (0.7 mA; FC-HS), the same used during training. Contextual fear memory and fear sensitization were evaluated 2 days and 4 weeks after high footshock (HS) treatment. A group of FC mice left undisturbed after training was considered as control in contextual and fear sensitization tests (Figure 7A).

Statistical analysis (one- and two-way ANOVAs: *F* > 5; *P* < 0.05) showed significant differences between FC and FC-HS mice in both contextual memory (Figure 7B) and fear sensitization (Figure 7C) test. In particular, HS treatment increased freezing levels in both tests, ruling out the hypothesis that PT treatment affects fear expression through a habituation process.

Alternatively, it is possible to envisage that the repeated footshock administration during PT treatment could increase the expectancy about the occurrence of US, minimizing the aversive value of context and then reducing fear expression. If so the administration of random footshock during PT treatment should prevent the reduction of fear expression. Fear-conditioned mice were exposed to a PT treatment (10 days) with a random PT administration (on day 1,3,7,9,10) 3 weeks after training (FC-PT_random_) and then submitted to contextual memory and fear sensitization tests. A group of FC mice was considered as control (Figure 7D). Statistical analysis showed significant differences (one- and two-way ANOVAs: *F* > 4; *P* < 0.05) between the two groups of mice in both contextual memory (Figure 7E) and fear sensitization (Figure 7F) tests, with FC-PT_random_ mice showing the lower levels of freezing. These results rule out the possibility that the effects of PT treatment on the reduction of fear expression are due to shock expectancy.

## Discussion

Fear memory after a trauma could induce emotional dysregulation due to the continuous re-experiencing of trauma reminders. Notably, a traumatic experience induces the formation of an associative memory and a nonassociative sensitization (Kamprath and Wotjak, 2004; Siegmund and Wotjak, 2007a). Sensitized fear is causally involved in the onset of abnormal behaviors characterizing anxiety disorders such as PTSD (Siegmund and Wotjak, 2007a; Costanzi et al., 2011).

Here we found that repeated exposure to a pain threshold footshock during contextual memory retrieval (PT treatment), but not a standard extinction procedure, is able to reduce anxiety levels, social withdrawal and learning deficits in a mouse model of learned fear. Moreover, such a treatment is effective in preventing the return of fear due to spontaneous recovery and reinstatement. Interestingly, contextual memory is not erased by the PT treatment, suggesting that this new approach is able to spare the adaptive value of fear memories.

Consistently, we found that amygdala activity is downregulated by the PT treatment. In line with findings showing the role of amygdala in fear expression and in the onset of anxiety behaviors (Beck and Fibiger, 1995; Ehrlich et al., 2009; Knapska and Maren, 2009; Poulos et al., 2009; Ciocchi et al., 2010; Haubensak et al., 2010; Tye et al., 2011; Mahan and Ressler, 2012; Pare and Duvarci, 2012), we found that the amount of freezing shown by both treated and no treated mice positively correlated with the number of activated neurons (c-fos+) in this region. It is noteworthy that the downregulation exerted by the PT treatment, as well as the correlation between freezing and c-fos+ neurons, are more evident in the medial part of the central nucleus (CeM), which plays a major role in regulating the expression of fear.

Which is the mechanism that allows PT treatment to be effective in reducing fear expression?

A possible explanation is that PT treatment is able to induce a re-evaluation of the US. That is, animals give a new value to the US and the acquisition of this new value—namely, its reduced aversiveness—is able to dampen the expression of fear.

According to a conditioning model of acquired fear proposed by Davey (1989), “the CS, through its learnt association with the US, comes to elicit a cognitive representation of the US. This representation of the US is then evaluated, and the resultant evaluation determines the strength, and in some cases the form, of the CR [conditioned response]” (p. 523). Thus, factors that modify the representation of the US can affect the CR independently from any changes in the associative strength between CS and US (Davey, 1989). For instance, in a seminal work by Rescorla and Heth (1975), post-conditioning reduction in US intensity was able to decrease the CR in rats (Rescorla and Heth, 1975). Similarly Kehoe and White (2002) reported that reductions in US intensity yielded a proportional reduction in the level of CR in rabbits (Kehoe and White, 2002). In humans, a post-conditioning US inflation procedure, in which a series of US became gradually stronger in intensity, determined an increase in CR (White and Davey, 1989); conversely, US deflation decreased the CR (Hosoba et al., 2001).

In agreement, we found that PT treatment is able to reduce nonassociative fear sensitization (which is independent from CS-US contingency, Kamprath and Wotjak, 2004; Costanzi et al., 2011) and it is effective also if administered in a context different from that used for the original conditioning (that is, in the absence of the CS). Both findings indicate that the effect of PT treatment is independent from CS-US association and strongly suggest that a US re-evaluation process can occur.

In order to verify that PT treatment effectively induced a re-evaluation of the US we performed lOFC lesions. Converging lines of evidence indicate a role for lOFC in processing information about the US value (Delamater, 2007). Indeed, animals with lOFC lesions do not show reduction of conditioned responses in devaluation paradigms in which an appetitive US is paired with drug-induced illness or satiation (Gallagher et al., 1999; Pickens et al., 2003; Poulos et al., 2009; West et al., 2011; van den Hout et al., 2013).

The results show that lOFC lesions block the beneficial effects of PT treatment on fear reduction and corroborate the hypothesis that PT treatment acts through a re-evaluation process (namely, US devaluation).

However, other plausible explanations can be considered. One possibility is that repeated exposure to US during the PT treatment could induce a habituation process, which also reduces the conditioned response by decreasing the salience of US representation (Rescorla, 1973). Alternatively, it is possible that repeated exposure to the US could reduce fear by increasing the expectancy of the aversive stimulus (Marcia et al., 1969; Huh et al., 2009). These hypotheses, however, were both ruled out by the results of control experiments we carried out to specifically address them.

From a clinical point of view, a number of case histories have been reported which illustrate the involvement of US inflation processes in the etiology of a variety of anxiety disorders (Davey et al., 1993). In particular, a man exposed to an event with relatively minor consequences developed PTSD symptoms after he was verbally informed about the actual danger he was exposed to on that occasion, suggesting that the US re-evaluation—in which the original US representation was changed in an aversive US representation (inflation)—could elicit the onset of anxiety-related responses (Davey et al., 1993).

More recently, Heir et al. (2009) reported that the PTSD symptom severity was related to recall amplification of the traumatic experience in Norwegian people exposed to the 2004 Tsunami, which also suggests that the inflation of trauma could increase traumatic memories and then the PTSD severity (Greenberg and Wessely, 2009).

Considering these clinical reports, we envisage that if trauma inflation (that is, the inflation of US representation) increases traumatic memories then trauma deflation could reduce traumatic memories and the severity of PTSD symptoms.

This idea has therapeutic implications. There is evidence showing that Eye Movement Desensitization and Restructuring (EMDR) and Cognitive Behavioral Therapy (CBT) are effective treatments for PTSD symptoms (Otte, 2011), although controversy about how these therapeutic approaches work continues to exist (Greenberg and Wessely, 2009; Otte, 2011; Jeffries and Davis, 2013; van den Hout et al., 2013; Zantvoord et al., 2013). Based on our results, we speculate that such therapeutic strategies might owe their efficacy to a process of cognitive re-evaluation of the US intensity, which is able to deflate the internal representation of the US (US devaluation).

This seems to be supported by the results obtained in laboratory studies on healthy humans submitted to a fear conditioning paradigm in which verbally transmitted information about a less intense US in future presentations was sufficient to weaker CR (Davey, 1983, 1987, 1989). In this context, we predict that the US devaluation should reduce the nonassociative component of traumatic memory (de-sensitization), sparing the memory for CS-US association.

In conclusion, we have shown that the re-evaluation of an aversive experience in FC mice restores an efficient fear regulation, by attenuating the expression of fear memory, without erasing it. This new approach spares the adaptive value of fear memory but mitigates the abnormal behavioral outcomes of a traumatic experience, paving the way towards new models suitable to test novel therapeutic approaches effective in treating anxiety disorders such as PTSD.

## Author contributions

Marco Costanzi designed the experiments, analyzed and interpreted the data, and wrote the first draft of the manuscript; Daniele Saraulli contributed to data interpretation and the final version of the manuscript; Sara Cannas collected and analyzed the data, contributed to the experimental design and the final version of the manuscript; Francesca D’Alessandro contributed to data collection and analysis; Fulvio Florenzano contributed to the interpretation of immunohistochemical data and confocal microscopy; Clelia Rossi-Arnaud and Vincenzo Cestari contributed to data interpretation, and the final version of the manuscript. All authors discussed the results and approved the final version of the manuscript.

## Conflict of interest statement

The authors declare that the research was conducted in the absence of any commercial or financial relationships that could be construed as a potential conflict of interest.
